# Special Issue for the School of Materials Science and Engineering at Southeast University

**DOI:** 10.34133/research.0519

**Published:** 2024-10-29

**Authors:** ZhengMing Sun

**Affiliations:** School of Materials Science and Engineering, Southeast University, Nanjing, P.R. China.

The School of Materials Science and Engineering at Southeast University is nestled along the scenic Jiulong Lake in Nanjing, south of the Yangtze River and west of the Zhongshan Mountains. As early as 1928, Southeast University (then known as National Central University) introduced undergraduate majors in engineering materials, as well as casting and forging materials. To advance materials science, the university established the Department of Materials Science and Engineering in December 1984, building upon the foundations of metal materials and heat treatment. Over time, majors such as Civil Engineering Materials from the Civil Engineering Department and Advanced Materials Processing from the Mechanical Engineering Department were integrated into this new department. In 2006, the department evolved into the School of Materials Science and Engineering, and in 2017, its Materials Science discipline was recognized as a “Double First-Class” national initiative.

Currently, the school encompasses 4 departments: Metal Materials, Civil Engineering Materials, Material Processing Engineering, and Advanced Functional Materials. It also offers a doctoral program and postdoctoral programs in the first-class discipline of Materials Science and Engineering, covering 3 second-level disciplines—Material Physics and Chemistry, Material Processing Engineering, and Materials Science. Additionally, it hosts a dedicated doctoral program in the second-level discipline of Civil Engineering Materials. The undergraduate program in Materials Science and Engineering is a provincial key major and a national signature program, while New Materials and Applications is a dominant key discipline, and Materials Science is a provincial key subject.

The School prides itself on an exceptional team, led by 2 academicians of the Chinese Academy of Engineering and featuring 22 national-level talents, including Changjiang Scholars Distinguished Professors and recipients of the National Science Fund for Distinguished Young Scholars. Among the 93 full-time faculty members, the majority have received international education or training.

The School also boasts numerous research and teaching platforms, such as the Jiangsu Collaborative Innovation Center for Advanced Civil Engineering Materials, Jiangsu Key Laboratory of Advanced Metal Materials, Jiangsu Key Laboratory of Civil Engineering Materials, and Southeast University’s National Metrology-Certified Analysis and Testing Center. Other key facilities include the Jiangsu Experimental Teaching Demonstration Center and the Innovation Platform for Materials Science Graduates.

Over the past 40 years, the School has played a leading role in hundreds of significant projects, including the National Basic Research Program (973), the National High-tech Research and Development Program (863), and projects supported by the National Natural Science Foundation of China. Its contributions to major national initiatives, such as the Shenzhou Spacecraft and the Three Gorges Dam, have been substantial. The School has been awarded 1 second prize for National Technological Inventions and 6 second prizes for National Scientific and Technological Progress, in addition to more than 30 provincial Science and Technology Progress Awards.

As a strong advocate of international collaboration, the School has established long-term partnerships with many prestigious universities worldwide, fostering cooperation in education, research, academic exchanges, and talent development.

